# EMPOWERING BETTER END-OF-LIFE DEMENTIA CARE (EMBED-CARE): A DIGITAL CLINICAL DECISION SUPPORT FRAMEWORK

**DOI:** 10.1093/geroni/igad104.1810

**Published:** 2023-12-21

**Authors:** Nathan Davies, Elizabeth Sampson, Tofunmi Aworinde, Juliet Gillam, Charlotte Kenten, Bethan Phillips, Catherine Evans, Clare Ellis-Smith

**Affiliations:** University College London, London, England, United Kingdom; University College London, London, England, United Kingdom; King’s College London, London, England, United Kingdom; King’s College London, London, England, United Kingdom; University College London, London, England, United Kingdom; University College London, London, England, United Kingdom; King’s College London, London, England, United Kingdom; King’s College London, London, England, United Kingdom

## Abstract

People with dementia experience a variety of unmet palliative care needs. There is a need to empower people with dementia, family carers and professionals, to be able to better assess and monitor needs, and manage symptoms. We aimed to co-design and evaluate acceptability, feasibility and implementation of an integrated model of palliative dementia care to support holistic assessment, decision making and management of needs for those living in community and residential care (EMBED-Care Framework).We used a co-design approach, using framework analysis to synthesize data from evidence reviews, routine data, and cohort study, we presented findings to a series of co-design workshops, to produce the Framework. The Framework consists of a holistic assessment; priority setting; and decision support using clinical decision support tools, delivered on an app. The holistic assessment identifies areas of concern and identify priorities of care, through a process of shared decision making. Each concern is linked to a decision support tool to support decision making and to address priorities. App alerts and reminders communicate concerns among teams and carers. We are testing the Framework in a 6-month feasibility study with two residential homes and two community settings in England. We are collecting outcomes including decisional conflict, satisfaction with care, and unmet needs. We are interviewing all users to explore acceptability and implementation. We will present development of the EMBED-Care Framework and preliminary findings from our feasibility study, focusing on the acceptability and lessons for implementation which can be applied to the wider dementia community and similar interventions.

